# A Novel Five-Gene Signature for Prognosis Prediction in Hepatocellular Carcinoma

**DOI:** 10.3389/fonc.2021.642563

**Published:** 2021-07-16

**Authors:** Lisa Su, Genhao Zhang, Xiangdong Kong

**Affiliations:** ^1^ Department of Genetic and Prenatal Diagnosis Center, The First Affiliated Hospital of Zhengzhou University, Zhengzhou, China; ^2^ Department of Blood Transfusion, The First Affiliated Hospital of Zhengzhou University, Zhengzhou, China

**Keywords:** HCC (hepatocellular carcinoma), prognosis, signature, risk score, overall survival

## Abstract

Hepatocellular carcinoma (HCC) has been a global health issue and attracted wide attention due to its high incidence and poor outcomes. In this study, our purpose was to explore an effective prognostic marker for HCC. Five cohort profile datasets from GEO (GSE25097, GSE36376, GSE62232, GSE76427 and GSE101685) were integrated with TCGA-LIHC and GTEx dataset to identify differentially expressed genes (DEGs) between normal and cancer tissues in HCC patients, then 5 upregulated differentially expressed genes and 32 downregulated DEGs were identified as common DEGs in total. Next, we systematically explored the relationship between the expression of 37 common DEGs in tumor tissues and overall survival (OS) rate of HCC patients in TCGA and constructed a novel prognostic model composed of five genes (AURKA, PZP, RACGAP1, ACOT12 and LCAT). Furthermore, the predicted performance of the five-gene signature was verified in ICGC and another independent clinical samples cohort, and the results demonstrated that the signature performed well in predicting the OS rate of patients with HCC. What is more, the signature was an independent hazard factor for HCC patients when considering other clinical factors in the three cohorts. Finally, we found the signature was significantly associated with HCC immune microenvironment. In conclusion, the prognostic five-gene signature identified in our present study could efficiently classify patients with HCC into subgroups with low and high risk of longer overall survival time and help clinicians make decisions for individualized treatment.

## Introduction

Hepatocellular carcinoma (HCC) has been a global health issue and attracted wide attention due to its high incidence and poor outcomes (1). It is reported that HCC results in nearly 850,000 new cases and more than 600,000 death every year (2), which seriously increases the disease burden in the worldwide. In China, HCC appears to be the most common pathological type of tumors and the major cause of cancer deaths (3). Moreover, the incidence and mortality are still escalating according to the increased alcohol abuse, cirrhosis, aflatoxin exposure, diabetes, metabolic syndrome and obesity (4–9). With the rapid developments in therapeutic strategies including liver resection, liver transplantation, radiofrequency ablation, embolization therapy and immune checkpoint inhibitors, HCC patients could access potential treatment strategies at early and intermediate stages of HCC prognosis (10). However, over half of HCC patients are in advanced stage when diagnosed, only 15% of which are suitable for curative therapies, and the five-year survival rate remains very low, no more than 20%, according to lacking of biomarkers for diagnosis at early stage and the high frequency of recurrence (11, 12). Consequently, it is necessary for us to seek novel prognostic markers for improving the poor outcomes of HCC patients.

Considering the widely exploited next-generation sequencing technology in life sciences, integrating prognosis-related gene signature shows a great advantage in the prediction of HCC prognosis by using TCGA and GEO program. In our current study, a five-mRNA signature associated with overall survival (OS) rate of HCC patients was established, and the robustness of which was externally validated by ICGC dataset and another independent cohort. We explored the association between the prognostic signature and invasion of immune cells in HCC at the same time. Initial construction of the five-gene signature for patients with HCC will help clarify the underlying mechanism of HCC, accurately predict HCC prognosis and make a meaningful contribution to therapeutic strategies.

## Materials and Methods

### Public Patient Datasets and Identification of Differentially Expressed Genes (DEGs)

The mRNA expression data and clinical characteristics of patients with HCC from seven publicly available datasets including TCGA-LIHC and GTEx, GSE25097, GSE36376, GSE62232, GSE76427, GSE101685 and ICGC (LIRI-JP) were incorporated into our study. After log2 transformed and quantile normalized, the mRNA expression data were calculated by mean expression when more than one probe were detected. The “LIMMA” package was used to screen common DEGs between normal and tumor samples in the five databases from GEO with a cut-off criterion of adjust *P* value <0.05 and |log_2_FC|>1. Then “edgeR” package was used to obtain DEGs from TCGA-LIHC and GTEx cohort with the same cut-off criterion.

### Signature Identification and Risk-Score Model Construction

After common DEGs were screened from GEO and TCGA-LIHC datasets, the association between common DEGs and OS rate in TCGA-LIHC cohort was calculated by a univariable Cox proportional analysis. Then LASSO-Cox regression method with the “glmnet” package was performed to determine significant prognosis-related DEGs. The stepwise Cox regression analysis was used to evaluate the above prognosis-related genes and establish a prognostic signature. Risk score was finally established based on the basis of linearly combining the formula below with the mRNA expression level multiplied the multivariate Cox regression coefficient (β) model. Risk score = (β_mRNA1_ × mRNA1) + (β_mRNA2_ × mRNA2) +…+ (β_mRNAn_ × mRNAn). We stratified patients in TCGA database into high-risk or low-risk score subgroups due to optimal risk score threshold determined by R package “survival” and “survminer”. The predictive power and independence of the prognostic signature in TCGA were assessed by ROC analysis, Kaplan-Meier survival analysis and Cox proportional hazards regression analysis. The genomic alterations including mutations and putative copy-number alterations of genes in the signature were analyzed on the open platform of cBioPortal (http://www.cbioportal.org/) (13). The mRNA and protein expression of genes in the signature were explored in Gene Expression Profiling Interactive Analysis (GEPIA) (14) (http://gepia.cancer-pku.cn/) and Human Protein Atlas (HPA, www.proteinatlas.org), respectively. Furthermore, the expression of five genes in HCC cell lines were explored in Cancer Cell Line Encyclopedia (CCLE) (15) (https://portals.broadinstitute.org/ccle).

### Functional Analysis

The mRNAs positively and negatively correlated with the prognostic risk score in TCGA-LIHC dataset are calculated by Pearson correlation analysis (|R|>0.5 and *P*<0.001). GO and KEGG pathway analysis were used for functional annotation of the positively and negatively correlated genes by using R package “clusterProfiler”.

### Clinical Specimens and Quantitative Real-Time PCR (qRT-PCR) Analysis

Previous collected 59 fresh frozen tissues from HCC patients were selected as an independent validation cohort (16). qRT-PCR was used to detect the mRNA levels of the five gene (17), after the relative mRNAs expression levels were normalized to *β*-ACTIN and log_2_ transformed. Primer sequences are showed in **Table S1**.

### External Validation of the Five-mRNA Signature

ICGC dataset was downloaded from the ICGC portal (https://dcc.icgc.org/projects/LIRI-JP). Risk score of patients with survival data in ICGC and our independent cohort was calculated with above formula, and we stratified patients into high-risk or low-risk score subgroups. Then predictive power and independence of the prognostic model were evaluated in the two cohorts.

### Calculation of Immune Status and Immune Infiltrates Analysis

To assess the immune status of each sample, ESTIMATE algorithm was applied to estimate stromal and immune cells calculated from the TCGA cohort, measuring stromal levels (stromal scores) and degree of immunocyte infiltration (immune scores) (18). The association between prognostic risk score and immune, stromal scores were analyzed by Pearson correlation analysis. Furthermore, CIBERSORT algorithm, a kind of deconvolution algorithm based on gene expression, was applied to assess the cell composition of different tumor-infiltrating immune cells (TIICs) and to further examine the relationship between the prognostic risk scores and the immune microenvironment in TCGA database (19) (http://cibersort.stanford.edu/). Finally, the predictive ability of significantly changed TIIC was assessed by Kaplan-Meier survival analysis.

### Statistical Analysis

ROC curve analysis and Kaplan-Meier survival analysis were performed to assess the prediction performance of OS rate with R software (Version 4.0.3). Cox proportional model was performed to analyze relationship between prognostic signature and OS rate, together with other clinical features including age, gender, grade, TNM stage, vascular invasion, tumor status, cirrhosis, hepatitis virus infection and Child-pugh scores. Clinical characteristics of HCC patients in TCGA, ICGC and clinical validation cohorts were showed in **Table S2**. Results were considered statistically significant when P value <0.05.

## Results

### DEGs Identification in Patients With HCC

**Figure 1:**
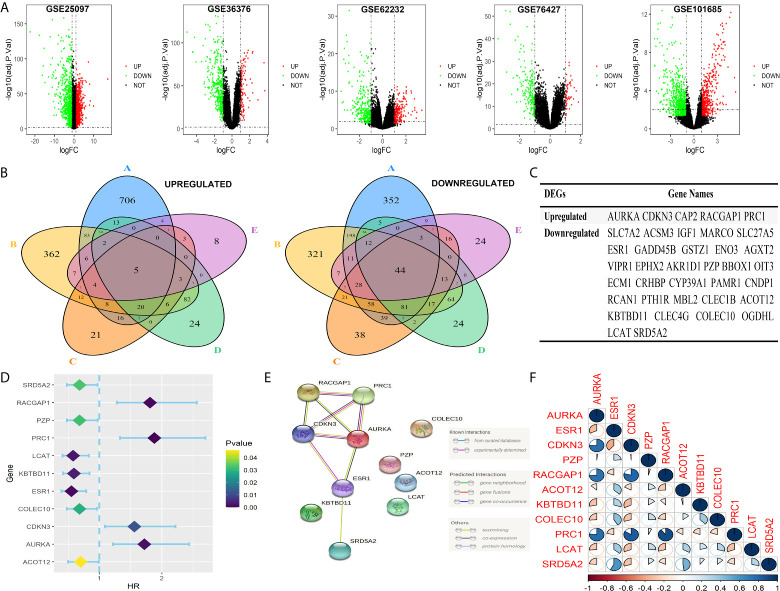
Identification of the common prognosis-related DEGs in five GEO and TCGA cohorts. **(A)** Volcano plots of DEGs in five GEO cohorts. DEGs were screened with a cut-off criterion of adjust *P* value <0.05 and |log_2_FC|>1. **(B)** The 49 overlapping genes were changed in tumor samples in five GEO cohorts. **(C)** Identification of 37 common DEGs in five GEO and TCGA cohorts. **(D)** Forest plots exhibited 11 prognosis-related DEGs to the results of univariable Cox analysis. **(E)** PPI network showing interactions among 11common DEGs. **(F)** The correlation of the 11 common DEGs.

DEGs in the five GEO datasets was screened with a cut-off standard of *P* value <0.05 and |log_2_FC|>1 first as shown in **Figure 1A**. Five upregulated and 44 downregulated genes were identified as common DEGs in the five GEO datasets (**Figure 1B**). When considering DEGs from TCGA-LIHC and GTEx cohort with the same cut-off criterion, 37 genes (5 upregulated and 32 downregulated, **Figure 1C**) were identified as common DEGs in both GEO, TCGA-LIHC and GTEx databases and were further analyzed.

### Establishment of a Prognosis-Related Signature in TCGA

**Figure 2:**
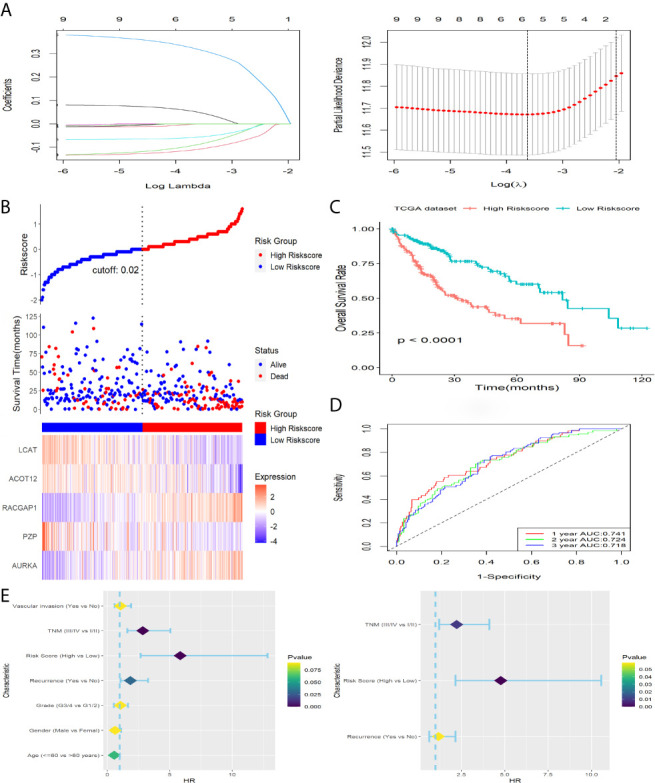
Construction and survival analysis of this five-gene signature in TCGA dataset. **(A)** Adjust parameter selection in LASSO-Cox analysis *via* 10 Cross-validation. **(B)** Distribution of risk score, OS status as well as gene expression patterns. **(C)** Kaplan-Meier survival plot. **(D)** ROC analysis of the signature in predicting1, 2, 3 years OS rate. **(E)** Forest plot showed results of univariable (left) and multivariable (right) Cox analysis on OS rate.

**Figure 3:**
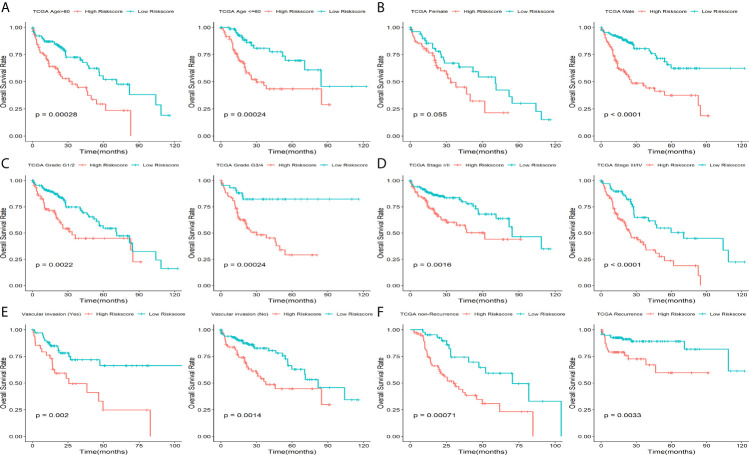
Prognostic significance of this five-gene signature in TCGA. Kaplan-Meier plot for HCC patients with different **(A)** age, **(B)** Gender, **(C)** Grade, **(D)** TNM stage, **(E)** vascular invasion status and **(F)** recurrence status.

**Figure 4:**
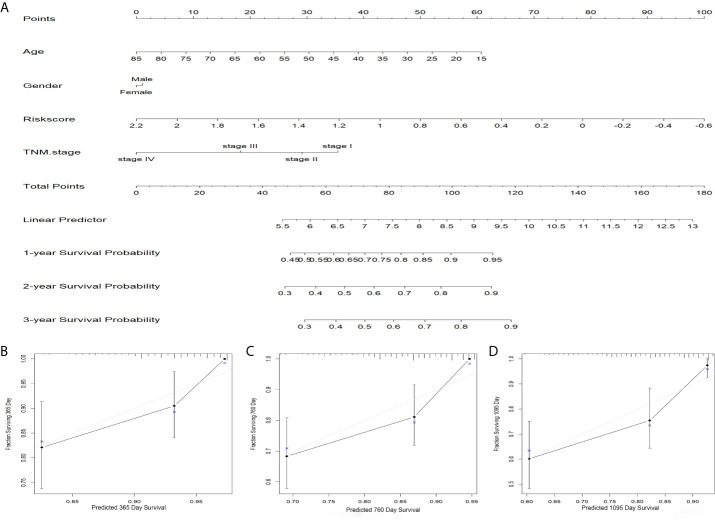
Predicted significance of signature verified in nomogram model. **(A)** A nomogram combining the five-gene signature. **(B–D)** The calibration plots for 1, 2, 3 years survival probabilities.

Eleven of the thirty-seven common DEGs were significant related to OS rate of patients with HCC in TCGA as shown in **Figure 1D**. The interworking network showed that ESR1, AURKA and CDKN3 were the central genes (**Figure 1E**) and the relevance of the eleven genes is shown in **Figure 1F**. Then significant prognostic genes among the 11 genes above were selected by performing the LASSO-Cox regression model based on the minimum value of λ and a prognostic five-gene signature was finally identified *via* a stepwise Cox proportional model (**Figure 2A**). Risk score = (0.0996 × AURKA) – (0.1421 × PZP) + (0.3809 × RACGAP1) – (0.0742 × ACOT12) – (0.1438 × LCAT). To determine whether there is a multicollinearity problem among five genes (AURKA, PZP, RACGAP1, ACOT12 and LCAT) expression and clinicopathological features, collinearity statistics was performed with a cut-off standard of tolerance >0.1 and variance inflation factor (VIF) <10. In fact, no multicollinearity problem exists as shown in **Table S3**. Risk score of patients in TCGA was calculated with the above formula, and patients were stratified into high-risk or low-risk subgroups with an optimal risk score threshold (**Figure 2B**). Result of Kaplan-Meier survival analysis revealed patients with higher risk score were significantly relevant to poor OS rate (**Figure 2C**). Result of ROC analysis revealed that this signature had a good prognostic performance, and the AUCs were 0.741, 0.724, 0.718 at 1-, 2-, 3-year, respectively (**Figure 2D**). Furthermore, we explored prognostic performance of the five-gene signature in patients with different clinical features including age, vascular invasion, grade, recurrence, TNM stage and gender, the results of which revealed that higher risk scores had a statistically relationship with shorter OS time in different clinical subgroups (**Figure 3**). Finally, statistically significant variables obtained from univariable Cox regression analysis were input into multivariate Cox regression analysis, and the results revealed that TNM stage, recurrence and risk score were statistically associated with OS, while TNM stage (HR = 2.228, 95%CI 1.208-4.108, *P* = 0.010) and risk score (HR = 4.773, 95%CI 2.157-10.561, *P* = 0.000) were independent prognostic factors in TCGA (**Figure 2E**). In order to discover the coefficient prediction efficiency of the prognosis-related signature, a nomogram model was established, the result of which showed that the nomogram with a C-index of 0.749 could help us provide a quantitative method for predicting the 1-, 2-, 3-year survival rate accurately (**Figure 4A**). The overlap of the forecasted probability and the actual probability of 1-, 2-, 3-year survival in calibration curve indicated a well agreement (**Figures 4B–D**).

### Verification of the Signature in ICGC Cohort

**Figure 5:**
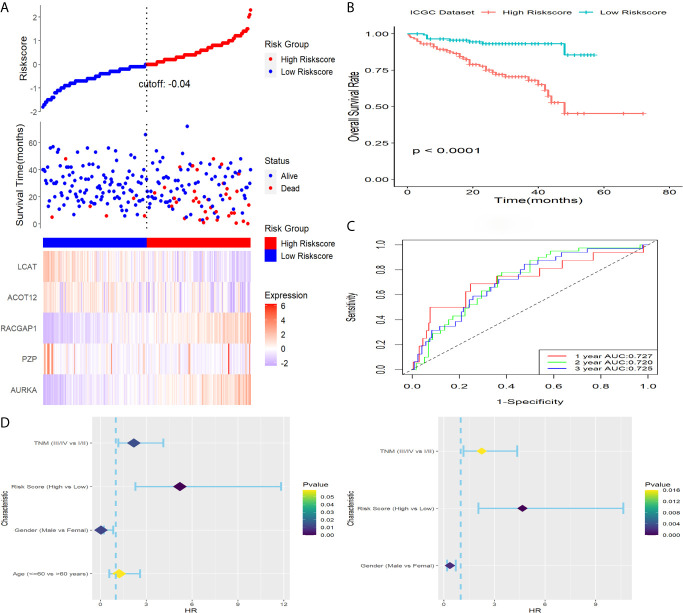
Verification of this signature in ICGC. **(A)** Distribution of risk score, OS status as well as gene expression patterns. **(B)** Kaplan-Meier survival plot. **(C)** ROC analysis of the signature in predicting1, 2, 3 years OS rate. **(D)** Forest plot showed results of univariable (left) and multivariable (right) Cox analysis on OS rate.

**Figure 6:**
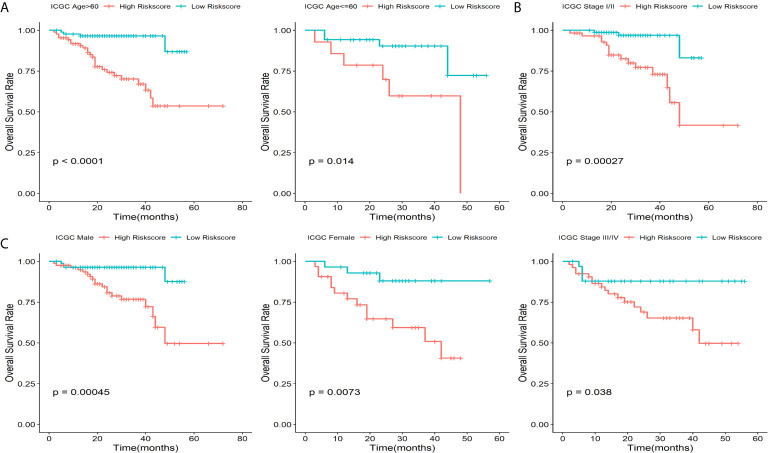
Prognostic significance of this five-gene signature in ICGC cohort. Kaplan-Meier plot for HCC patients with different **(A)** age, **(B)** TNM stage and **(C)** Gender.

To verify capability of the five-gene signature, ICGC dataset was downloaded as a validation cohort. The risk score of patients was calculated with the same formula, and we stratified patients into low-risk or high-risk subgroups (**Figure 5A**). Result of Kaplan-Meier survival analysis revealed that patients with higher risk score were prominently relevant to poor OS rate (**Figure 5B**). Result of ROC analysis revealed that the five-gene signature had a good prognostic performance, and the AUCs were 0.727, 0.720, 0.725 at 1-, 2-, 3-year, respectively (**Figure 5C**). Furthermore, the predictive performance of the five-gene signature was explored in patients with different clinical features such as age, gender and TNM stage, the results of which revealed that higher risk scores had a statistically relationship with shorter OS time in different clinical subgroups (**Figure 6**). Finally, statistically significant variables obtained from univariable Cox regression analysis were input into multivariate Cox regression analysis, and the results revealed that TNM stage, gender and risk score were statistically relevant to OS of HCC patients, furthermore, TNM stage (HR=2.246, 95%CI 1.162-4.339, *P* = 0.016), gender (HR=0.361, 95%CI 0.185-0.704, *P* = 0.002) and risk score (HR=4.662, 95%CI 2.044-10.632, *P* = 0.000) were independent prognostic factors for OS of patients with HCC in ICGC (**Figure 5D**).

### Verification of the Five-Gene Signature in a Clinical Cohort

**Figure 7:**
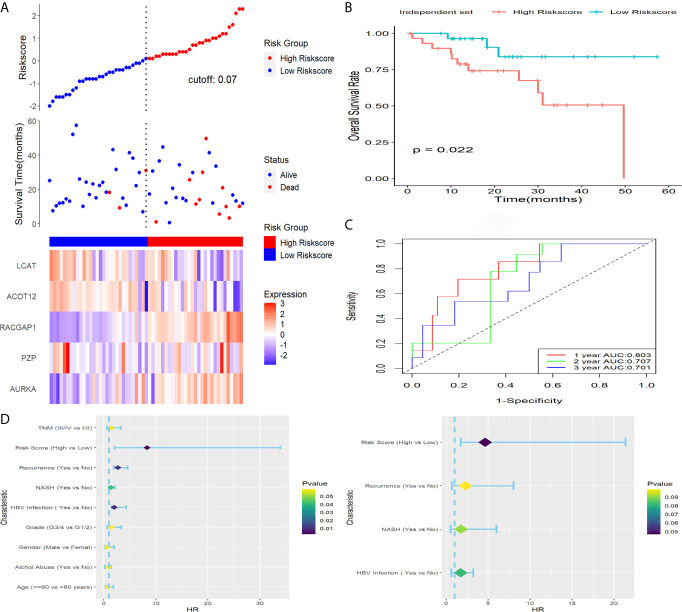
Verification of this signature in a clinical dataset. **(A)** Distribution of risk score, OS status as well as gene expression patterns. **(B)** Kaplan-Meier survival plot. **(C)** ROC analysis of the signature in predicting1, 2, 3 years OS rate. **(D)** Forest plot showed results of univariable (left) and multivariable (right) Cox analysis on OS rate.

To verify capability of the 5-gene signature in the prediction of OS rate in actual clinical practice, qRT-PCR analysis was performed in a clinical cohort. Risk score of patients was calculated with the same formula, and we stratified patients into high-risk or low-risk groups (**Figure 7A**). Result of Kaplan-Meier survival analysis revealed that patients with higher risk score were prominently relevant to poor OS rate (**Figure 7B**). Result of ROC analysis revealed that this signature had a good prognostic performance, and the AUCs were 0.803, 0.707, 0.701 at 1-, 2-, 3-year, respectively (**Figure 7C**). Furthermore, statistically significant variables obtained from univariable Cox regression analysis were input into multivariate Cox regression analysis, and the results revealed that HBV infection, risk score, NASH and recurrence were statistically relevant to OS of HCC patients, while the risk score (HR = 4.663, 95%CI 1.716-21.387, *P* = 0.047) was only independent prognostic factors (**Figure 7D**).

### Functional Analysis

**Figure 8:**
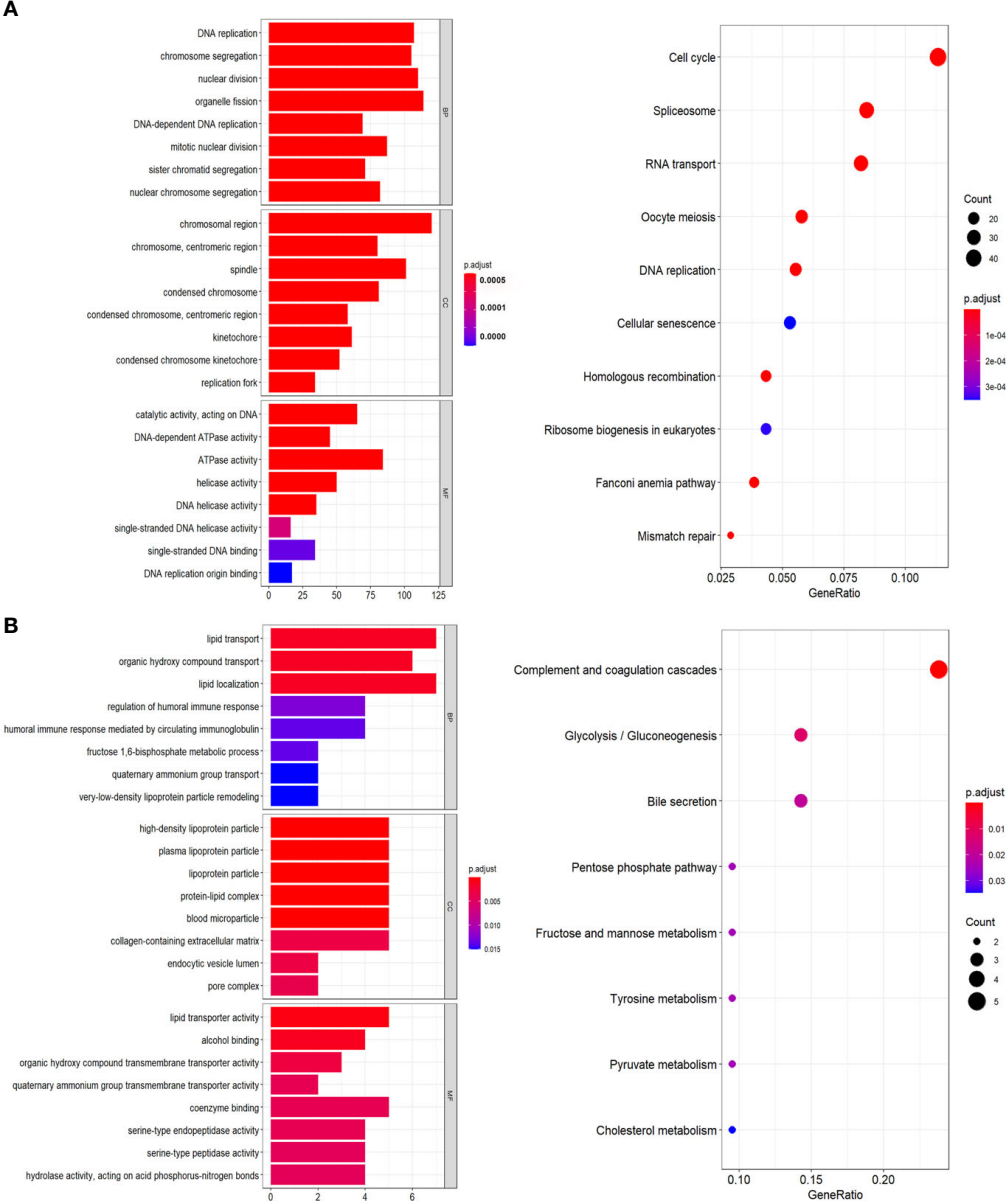
Functional annotation. **(A)** Genes positively related with signature. **(B)** Genes negatively correlated with signature.

According to the results of Pearson correlation analysis, screened 989 positively and 32 negatively correlated genes enriched GO terms could be classified into three functional clusters such as cell cycle and proliferation, lipid transport and localization, ATP metabolic process, while the mainly enriched pathway were cell cycle, spliceosome, complement and coagulation cascades (**Figure 8**).

### Genetic Alterations and Expression Level of the Genes in the Five-Gene Signature

366 samples with mutation and CNA data are available in cBioPort platform. AURKA, PZP, RACGAP1, ACOT12 and LCAT had missense mutation in 0.3%, 2.4%, 1.3%, 1.3%, 0.8% of HCC samples, respectively. Besides, AURKA, PZP, ACOT12 and LCAT had amplification in 1.6%, 0.3%, 0.3%, 0.3% of HCC samples, respectively. No putative copy-number alterations were found for RACGAP1. Amplification is the most common form of genetic alteration (**Figure S1A**). Additionally, the mRNA expression levels of the five genes differed significantly between tumor and normal specimens in GEPIA (**Figure S1B**), so as the protein expression levels of AURK, PZP and RACGAP1 in HPA (**Figure S1C**). Unfortunately, ACOT12 and LCAT were not found in HPA. Furthermore, the expression levels of these five genes were significantly different between HCC cell lines in CCLE (**Figure S1D**).

### Association Between Risk Score and Infiltration of Immune Cells in TCGA

**Figure 9:**
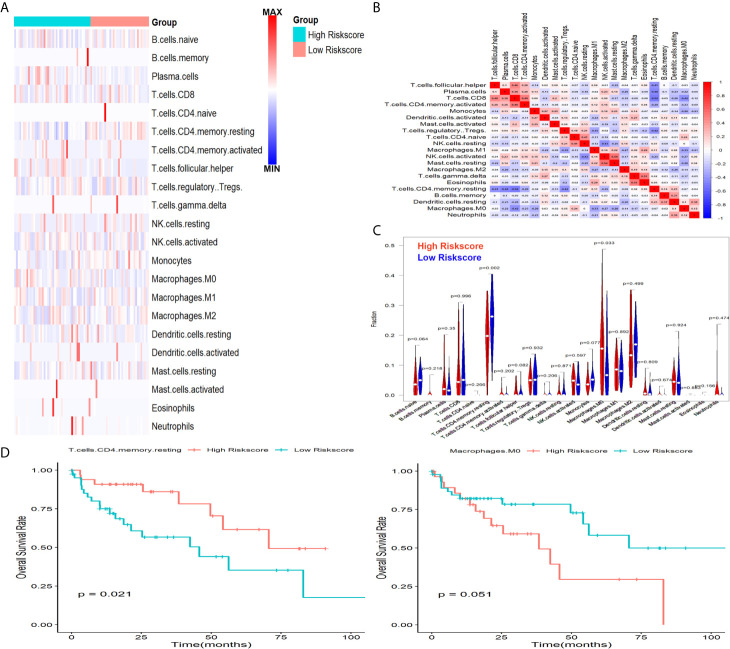
Associations between signature and infiltration of immune cells in HCC. **(A)** The heatmap showed the abundance of 22 TIICs in two subgroups. **(B)** The correlations of 22 TIICs in TCGA cohort. **(C)** The changed abundance of Macrophages.M0 and resting memory CD4 T cells. **(D)** Lower abundance of resting memory CD4 T cells were significantly related to poor OS.

On the strength of ESTIMATE algorithm, the prognostic risk scores have a significant association with stromal scores, but not immune scores, and samples in high-risk group had lower stromal scores when compared with samples in low-risk group (**Figure S2**), indicating that the prognostic risk scores had a close connection with tumor immune status. In the following, to understand how the prognostic risk scores reflected immune status of HCC, distinctions and correlations of 22 types of TIICs in two subgroups in TCGA were assessed *via* CIBERSORT (**Figures 9A, B**). According to the difference analysis, the proportion of macrophages.M0 was down-regulated in low-risk score group, while the proportion of resting memory CD4 T cells was up-regulated (**Figure 9C**). Furthermore, patients with decreased resting memory CD4 T cells were prominently relevant to poor prognosis (**Figure 9D**).

## Discussion

Although the TNM staging system and the prognostic scoring systems of American Joint Committee on Cancer are implemented to assess the prognosis of HCC patients, in current predictive methods each system cannot always be effective in predicting the prognosis according to the losing sight of different genetic and epigenetic backdrops of tumors (20). Herein, it becomes more important than ever before to explore effective biomarkers for improving the prognosis. In previous time, although many studies tried to clarify molecular mechanisms of HCC development, the achievement is still unsatisfied, because the molecular pathogenesis is extremely complex and heterogeneous (21). In this study, bioinformatics methods and expression profiling techniques have been performed to identify DEGs in patients with HCC. Five cohort profile datasets from GEO (GSE25097, GSE36376, GSE62232, GSE76427and GSE101685) were integrated with TCGA-LIHC and GTEx dataset, then 5 upregulated DEGs and 32 downregulated DEGs were identified as common DEGs in total and were further analyzed. Next, we systematically explored the relationship between the expression of 37 common DEGs in tumor tissues and OS rate in TCGA and constructed a novel prognosis-related model composed of five genes (AURKA, PZP, RACGAP1, ACOT12 and LCAT). Furthermore, the predicted value of the signature was validated in ICGC and another independent clinical samples cohort, which demonstrated that the signature had an excellent performance in the prediction of OS rate. What is more, it was an independent risk factor for patients with HCC when considering other clinical factors in the three cohorts. Finally, when we tried to explore the potential mechanisms, we found that the prognostic risk score had a close connection with stromal score, and patients in high-risk score group exhibited an increased abundance of Macrophages.M0 and decreased abundance of resting memory CD4 T cells in TCGA-LIHC dataset, which suggested that the signature was significantly associated with HCC immune microenvironment.

Most of the genes in our five-gene signature had been reported to participate in cancer development. AURKA was reported to act a pivotal part in abrogating G2 checkpoint induced by DNA damage in urothelial cell carcinoma (22, 23). Inhibition of AURKA could reduce the activity of poly (ADP-ribose) 1 level and promote non-homologous end joining repair (NHEJ) mechanisms in ovarian carcinoma cells (24). Blocking the β-catenin pathway and inhibiting AURKA activity at the same time may enhance antitumor response in adrenocortical cancer (25). AURKA was essential for mediating TGF-beta induced plasticity and chemoresistance in triple-negative breast cancer (26). Liu (27) found that knockdown of AURKA could lead to increased radiotherapy efficacy in human colorectal cancer. Moreover, overexpressed AURKA might promote hepatocellular carcinoma cell growth, adhesion and migration *in vitro (* 28). RACGAP1 has been identified as a hub gene and significantly associated with overall survival in patients with various cancer types, such as pancreatic ductal adenocarcinoma (29), gastric cancer (30), cervical cancer (31) and lung adenocarcinoma (32). Furthermore, Zhao has found that depletion of RACGAP1 could lead to mitotic catastrophe and massive cell death in esophageal squamous cell carcinoma (33). Kehan confirmed that RACGAP1 played an essential role in breast cancer metastasis by modulating ECT2-dependent mitochondrial quality control (34). Yong found that RACGAP1 overexpression was significantly related to poor prognosis in HCC patients, and could promote proliferation ability of HCC cells *via* inhibiting activation of the Hippo and YAP pathways (35). Chen found that ten hub genes associated with immune infiltration, including RACGAP1 and AURKA, could predict survival outcome in HCC *via* bioinformatics analysis (36). The down-regulated ACOT12 was significantly associated with poor diagnosis in metastatic HCC patients (37). ACOT12 could facilitate metastasis through epigenetic induction of epithelial-mesenchymal transition in HCC (38). Previous study found that LCAT was overexpressed in the sera of high-grade and lymph-node-positive breast cancer and could be a common plasma protein marker in aggressive breast cancer (39). Besides, LCAT was hypermethylated and decreased in HCC tissues (40), could act as a good biomarker at predicting HCC diagnosis, prognosis and recurrence (41), and it was confirmed in the later study by Long (42). As for PZP, in previous study we have confirmed that its depletion was significantly related to poor survival outcomes in HCC (16). All these indicated that AURKA, PZP, RACGAP1, ACOT12 and LCAT played important roles in the development of HCC and might be targets for immunotherapeutic intervention strategies in further.

In the present study, we identified a prognosis-related biomarker to stratify HCC patients and forecast the prognosis. When compared with previous signatures (43–48), some genes in the signature, such AURKA, LCAT and ACOT12, were also identified as hub genes in predicting survival outcomes of HCC patients, in agreement with our study. Additionally, the five-gene signature had some novelties although all of them could effectively predict the prognosis of HCC patients. Firstly, five GEO datasets, TCGA-LIHC and GTEx were incorporated into our study to screen common DEGs between normal and tumor samples, making the DEGs more reliable than those in previous research. Secondly, in order to ensure the clinical relevance, qRT-PCR analysis was performed to validate the signature in a clinical cohort after the signature was constructed in TCGA cohort. Thirdly, strong prognostic performance among different clinical characters made the five-gene signature more attractive for clinical implementation. Finally, the five-gene signature contains fewer genes to make it more easily to implement in comparison with previous signature. There is no denying that our present study existed certain limitations. The great heterogeneity of HCC and the mechanisms of post-curative recurrence might decrease the performance of the prognostic model. Moreover, the association between prognostic risk score and infiltration of immune cells maybe inaccurate when considering it is based on estimated tumor characteristics. In addition, multicenter randomized controlled studies combining mRNA, single-nucleotide polymorphism, CpG and long non-coding RNA are needed to investigate the five-gene signature in the future.

In conclusion, a prognostic five-gene signature was identified in our present study, which could efficiently classify HCC patients and help clinicians make decisions for individualized treatment.

## Data Availability Statement

The datasets presented in this study can be found in online repositories. The names of the repository/repositories and accession number(s) can be found in the article/**Supplementary Material**.

## Ethics Statement

The studies involving human participants were reviewed and approved by Ethics Committees of Zhengzhou University. The patients/participants provided their written informed consent to participate in this study.

## Author Contributions

GZ designed the study, downloaded and analyzed the data. LS wrote the manuscript. XK critically reviewed the manuscript. All authors contributed to the article and approved the submitted version.

## Conflict of Interest

The authors declare that the research was conducted in the absence of any commercial or financial relationships that could be construed as a potential conflict of interest.

## Supplementary Material

Supplementary Figure 1 Genetic alterations and expression level of genes in the prognostic significance. **(A)** The genetic alteration of the five genes in TCGA-LIHC. **(B)** The mRNA expression levels of the five genes in GEPIA. **p* < 0.05. **(C)** The protein expression levels of the five genes in HPA. **(D)**. The expression levels of the five genes in CCLE.

Supplementary Figure 2 Relationship between prognostic significance and immune status in TCGA. Correlation **(A)** and difference **(B)** analysis of prognostic risk scores with immune status.

Supplementary Table 1 The sequences of the qRT-PCR primers.

Supplementary Table 2 Clinical characteristics of HCC patients involved in TCGA, ICGC and the clinical cohort.
