# Supplementary figures and images for: A Novel Five-Gene Signature for Prognosis Prediction in Hepatocellular Carcinoma
Source: Front Oncol. 2021 Jul 16;11:642563. doi: 10.3389/fonc.2021.642563 (PMC8322700; doi:10.3389/fonc.2021.642563)

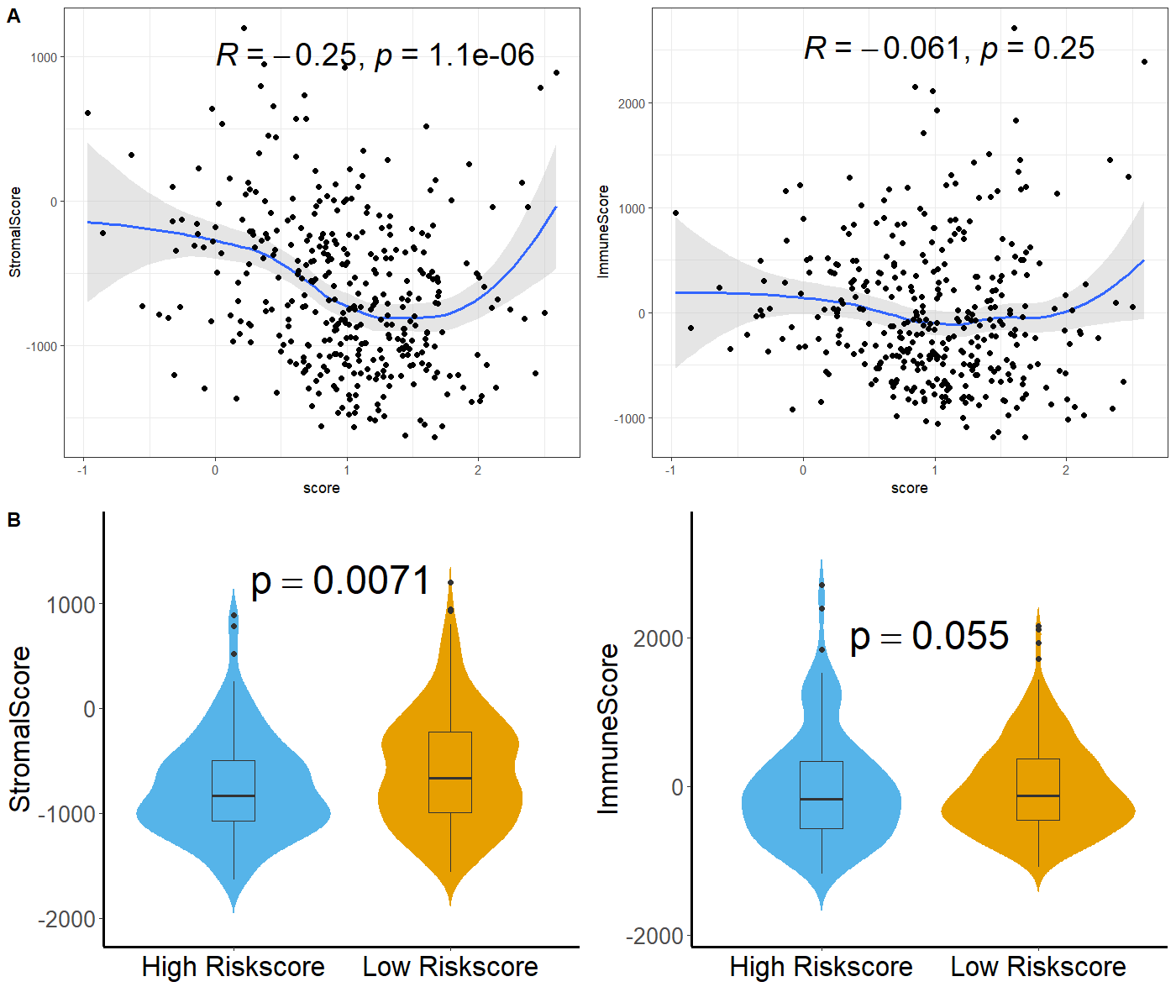

Supplement: Supplementary Figure 2 — Relationship between prognostic significance and immune status in TCGA. Correlation (A) and difference (B) analysis of prognostic risk scores with immune status. [file Image_2.png]
