# Supplementary material for: A Novel Five-Gene Signature for Prognosis Prediction in Hepatocellular Carcinoma
Source: Front Oncol. 2021 Jul 16;11:642563. doi: 10.3389/fonc.2021.642563 (PMC8322700; doi:10.3389/fonc.2021.642563)
Supplement: Supplementary Table 1 — The sequences of the qRT-PCR primers. [file Table_1.pdf]

Table. S1 The sequences of the qRT-PCR primers used in this study

Gene	Forward primer	Reverse primer
**AURKA**	GAGGTCCAAAACGTGTTCTCG	ACAGGATGAGGTACACTGGTTG
**PZP**	CACAGGAAACCGTCCTGCTT	ACTCAGCAACCACAGACTCAT
**RACGAP1**	TCCAATTTATCCAGTTGGCGAA	CTTCAGCTTAACATCCAGAGCA
**ACOT12**	GAGGAAGGAGCGGTTTCCAC	AGGCGGCTTGCAGAAATAGTA
**LCAT**	ACCTGGTCAACAATGGCTACG	TAGAGCAAGTGTAGACAGCCG
**β-ACTIN**	CGTGGGCCGCCCTAGGCACCA	TTGGCTTAGGGTTCAGGGGGG

Table S2 Clinical characteristics of HCC patients involved in the study

		TCGA cohort (N=365)	ICGC cohort (N=227)	Independent cohort (N=59)
**Gender**	**Male**	119	61	45
	**Female**	246	166	14
**Age**	**≤60 years**	173	49	27
	**>60 years**	192	178	32
**Grade**	**G1/2**	230		35
	**G3/4**	130		24
	**unknown**	5		
**TNM Stage**	**I/II**	254	140	42
	**III/IV**	87	87	17
	**unknown**	24	0	0
**Vascular Invasion**	**Yes**	106		
	**No**	205		
	**unknown**	5		
**Recurrence**	**With tumor**	122		29
	**Tumor free**	161		30
	**unknown**	82		0
**Cirrhosis**	**With**	68		6
	**Without**	141		53
	**unknown**	156		0
**HBV or HCV Infection**				
	**Yes**	149		41
	**No**	203		18
	**unknown**	13		0
**Child-Pugh**	**A**	216		57
	**B**	21		2
	**C**	1		0

**unknown**	127	0

Table. S3 The results of collinearity statistics

	**Tolerance**	**Variance Inflation Factor (VIF)**
**AURKA**	0.439	2.280
**PZP**	0.901	1.110
**RACGAP1**	0.415	2.412
**ACOT12**	0.839	1.192
**LCAT**	0.795	1.258
